# Factors predicting secondary school language course enrollment and performance among U.S. heritage speakers of Spanish

**DOI:** 10.3389/fpsyg.2022.1035716

**Published:** 2023-01-11

**Authors:** My V. H. Nguyen, Ellen J. Serafini, Jennifer Leeman, Adam Winsler

**Affiliations:** ^1^Department of Psychology, University of Houston, Houston, TX, United States; ^2^Department of Modern and Classical Languages, George Mason University, Fairfax, VA, United States; ^3^Department of Psychology, George Mason University, Fairfax, VA, United States

**Keywords:** heritage language learners, Spanish, language courses, language learning, language education, educational equity

## Abstract

**Introduction:**

While a growing body of research indicates that Spanish language courses can promote Spanish maintenance and lead to overall improved educational outcomes among heritage speakers, there is little empirical or longitudinal evidence of factors that shape their enrollment in Spanish language courses at the secondary level. To address this issue, the current study takes a large-scale, longitudinal approach to investigate rates of enrollment in secondary school (6th–12th grade) Spanish and other non-English language courses, as well as factors that predict heritage speakers’ enrollment and performance in non-English language courses.

**Method:**

We analyzed subsample data from the Miami School Readiness Project (MSRP), a large-scale, longitudinal study consisting of 17,341 heritage speakers of Spanish (47% female, 95.4% Hispanic/Latino, 82.8% received free/reduced-price lunch, and 18.3% with a disability) who were followed from 4 years old until the end of high school.

**Results:**

In general, Heritage speakers enrolled in Spanish language courses at a higher rate than other non-English language courses (52.2 and 25.3%, respectively). Enrollment patterns varied across different type of languages and grade level. Student-level factors including disability status, poverty status, early behavioral problems, and prior academic achievement significantly predicted students’ enrollment in Spanish and performance in non-English language courses.

**Discussion:**

Findings shed light on the long-term patterns of language study of this growing segment of the US school population with implications for future research and school policies that seek to improve heritage language learning and maintenance as well as equitable access to language education for language-minority students.

## Introduction

In the United States, speakers of languages other than English face many challenges in maintaining their home language and/or passing it on to their children. Among the most significant obstacles is the lack of sustained educational opportunities to build proficiency and academic literacy in those languages, which is linked to state and national language policies that promote English monolingualism (Cummins, 2005; Wiley and García, 2016; Ennser-Kananen and King, 2018; Fuller and Leeman, 2020). While local community-based schools historically have played a key role in supporting minority language maintenance and linguistic diversity (Fishman, 2001), since the early 20th century, the dominant language-in-education paradigm in the U.S. has been English-medium schooling. This policy is buoyed by pervasive English-only ideologies and discourses that portray proficiency in non-English languages as a threat to national unity and an impediment to the acquisition or mastery of English (Crawford, 2000; Wiley, 2000).

This lack of consistent educational support, coupled with a “language as problem” (Ruiz, 1984) orientation to language in policy and planning, contributes to the dominant pattern of a shift to English language dominance/use over time within immigrant families and the loss of the heritage language, typically by the third generation (Veltman, 1983; Fishman, 2001; Alba, 2004; Rumbaut, 2009). As the most commonly spoken non-English language in the U.S. with deep sociohistorical roots and continuous immigration patterns, some have suggested that Spanish might be more immune to language shift and loss than other minoritized languages. However, while research indicates that Spanish is sometimes maintained beyond the third generation, this is typically only in areas with a high density of Spanish-speakers and continued immigration, and it remains the exception rather than the norm (Alba, 2004; Mora et al., 2005; Villa and Rivera-Mills, 2009).

Heritage speakers of Spanish – that is, individuals who grow up in Spanish-speaking homes and who have some degree of proficiency in Spanish (Valdés, 2001) – face several hurdles within the K-12 U.S. public schooling system that limit their opportunities to maintain their home language and further develop their bilingual and bicultural identities. One key barrier is the persistent emphasis on some form of English-immersion schooling and lack of bilingual schooling options. The limited bilingual education programs that are available in the U.S. (Redford, 2018) are often subtractive in that they prioritize children’s acquisition and use of English and are designed to transition students to mainstream English classrooms as quickly as possible without support for the home language, which does not align with evidence that knowledge and skills transfer from the home language to the school language (Cummins, 2000).

On the other hand, dual language immersion (DLI) models are additive models designed to promote bilingualism, biliteracy, academic achievement, and intercultural competence among both majority-and minority-language children (Howard et al., 2018). A growing body of longitudinal empirical evidence demonstrates the superiority of additive DLI models in terms of promoting faster English acquisition and stronger academic achievement in the long-term for language-minority students (Thomas and Collier, 2002; Marian et al., 2013; Umansky and Reardon, 2014; Steele et al., 2017; Serafini et al., 2020). Interestingly, recent research also reports that being bilingual and being enrolled in bilingual education at the primary level may impact later language study. For instance, Nguyen and Winsler (2021) found that early bilingualism in kindergarten was a significant predictor of later foreign language course enrollment and performance in middle or high school, and this held true after controlling for demographic factors, school readiness skills, and early academic achievement. Another recent study found that both Spanish-speaking and non-Spanish-speaking students who were enrolled in DLI programs continued on to advanced language study at the secondary level with high levels of performance (Padilla et al., 2022).

In spite of this evidence demonstrating the benefits of additive bilingual programs, such programs remain few and far between. Although, thirty-nine states and the District of Columbia reported offering dual language education programs during the 2012–2013 school year with Spanish as the most commonly reported partner language followed by Chinese (Boyle et al., 2015), these programs serve just a small minority of English language learners. According to a nationally representative study of the 2010–2011 school year (Redford, 2018), only 8% of kindergarteners participating in English language programs received dual language education as the primary type of instruction while 60% received English as a Second Language instruction as the primary type of instruction. Moreover, 57% of kindergarteners in English language programs did not receive *any* academic instruction in their native language (Redford, 2018).

The dominance of subtractive models of English language instruction and the lack of school-based support for minoritized languages reflects the impact of dominant monolingual ideologies, including the staffing challenges that result from a lack of pre-service training for dual language teachers. Further, scholars have recently argued that many of the DLI programs that do exist have moved away from prioritizing the dynamic bilingualism and cultural identities of Hispanic/Latina/o/x and other minoritized communities and toward serving the needs of non-Hispanic White students and students from English-speaking and/or affluent homes (Valdez et al., 2016; Flores and García, 2017; Flores et al., 2021), thus under-serving minority-language populations.

Educational opportunities to study languages other than English as a subject become more common in the secondary school context middle school (6th–8th grade) and high school (9th–12th grade). However, such opportunities most often come in the form of “Spanish as a ‘foreign’ language” classes and are typically offered as an elective for a few hours per week. In a national report (American Councils for International Education, 2017), just 20% of the school-age population enroll in world/foreign language elective courses in a given school year (2014–2015 for this source). In other words, only around 1 in 5 U.S. public school students study a world or ‘foreign’ language at all, with an overwhelming majority of these enrolled in Spanish. Moreover, children in poverty and children of color are far less likely to have access to L2 language courses, thus providing them with less opportunity to develop multilingual skills and negatively impacting their chances for attending higher education (Darling-Hammond, 2001; Baggett, 2016). However, to date, large-scale research has not investigated which factors, such as socioeconomic status or home language, impact the language(s) that heritage speakers study in the secondary setting.

Importantly, despite the fact that a quarter of the public school-age population identifies as Hispanic/Latina/o/x (National Center for Education Statistics, 2015), the Spanish curricula in these “foreign” language courses are typically designed to meet the needs of monolingual second language (L2) learners exposed to Spanish exclusively in formal contexts, rather than draw on or develop the bilingual and bicultural knowledge, experiences, and abilities of students who acquired Spanish in home or community settings (Beaudrie et al., 2014; Parra, 2020; Leeman and Serafini, 2021). For example, heritage speakers tend to have strong listening comprehension and oral fluency skills with the ability to naturally converse about a range of daily topics. As a result, heritage speakers of Spanish may be bored in such classes or alternatively find them too difficult because they focus on metalinguistic knowledge more typical of L2 learners (Potowski, 2002). Moreover, Spanish courses often focus on so-called standard varieties spoken by monolingual elites, which may differ from the varieties that heritage speakers learn at home. Finally, there is a pervasive discourse that heritage speakers take Spanish to earn an “easy A,” which may impact their sense of belonging and deter them from studying it in a formal setting, sometimes leading them to choose a different “foreign” language (Leeman and Serafini, 2021).

This misalignment in curricular and learner needs, together with Civil-Rights-era calls for more equitable and inclusive educational policies and practices, spurred the development of the field of Spanish heritage language education (SHL; Valdés, 1981). In contrast to Spanish as a foreign language, SHL courses and programs start from the premise that students arrive in class with some prior knowledge of Spanish, whether receptive or productive knowledge. Heritage speakers’ linguistic and cultural knowledge and experiences are integrated into the curriculum (Beaudrie, 2015). Like Spanish as a foreign language course, some SHL courses also reproduce the standard language ideology, but appreciation of linguistic variation is increasingly seen as a key goal (Beaudrie et al., 2014), and in some cases, students’ critical awareness and understanding of relationships between language and power are also core objectives Leeman, 2005, 2018; Leeman and Serafini, 2016; Holguín Mendoza, 2018; Beaudrie and Loza, in press; Holguín Mendoza, 2022). Recent evidence has demonstrated that Spanish courses and programs designed specifically for heritage speakers are linked to improved educational and emotional outcomes as well as stronger language maintenance and student retention (Amezcua, 2019; Jang and Brutt-Griffler, 2019; Prada et al., 2021). While no comprehensive national survey data currently exist for heritage courses and program offerings or heritage student enrollment at K-12 level by state, research indicates that only a small minority of U.S. public school students have access to heritage language courses at the secondary level (Prada et al., 2021).

### Motivation for the current study

The predominance of English-dominant models of schooling in the U.S. and a lack of access to both bilingual education and heritage language courses and programs, coupled with the dominance of Spanish courses geared toward L2 learners potentially make it less likely that heritage speakers of Spanish will elect to study it at the secondary level and beyond. However, to date, little to no large-scale longitudinal research has investigated what factors impact heritage student enrollment and performance in Spanish or non-English language courses in middle school or high school. One recent study conducted in a community college setting sheds some initial light on this question in a community college context (Nagano et al., 2019). In their analysis of data from a nationwide survey of heritage student enrollment in heritage languages courses versus studying a third language (L3) in community college (*N* = 1,756), almost half, or 42%, identified as heritage speakers and slightly less than half of these students (45%) reported studying an L3. In contrast, slightly over half (55%) were studying their heritage language. The authors found that “the primary reason for HL speakers not studying their HL is the lack of modern language courses offered in their HL” (p. 324), particularly for less commonly taught languages. However, given that Spanish was the most frequently offered language at the community colleges included in the sample, this did not explain the relatively high number of Spanish heritage speakers who chose to study an L3 (36%) instead of Spanish (64%), which was linked to differences in type of motivation (integrative vs. instrumental).

While Nagano et al. (2019) study offers an initial look at this issue in higher education, there is no research exploring the language course enrollment patterns of Spanish heritage speakers in the K-12 setting or what factors predict whether students choose to study Spanish in middle school/junior high (grades 6–8) or high school (grades 9–12). In general, we know that students of color have less access to and are significantly underrepresented in world language courses (Baggett, 2016). However, there is “no known literature that has reported Latino/a student enrollment patterns” (p. 163). Exploring this question empirically could not only lead to a better understanding of the language enrollment patterns of heritage speakers, but also shed light on whether heritage speakers choose to study the heritage language, a third language, or none at all. While the examination of enrollment patterns cannot not tell us about their underlying motivations or motivational profiles (see Stewart-Strobelt and Chen, 2003; Thompson, 2017), such large-scale data would be useful for identifying “specific gateways for student enrollment, including policies regarding tracking, and school personnel that may make recommendations related to enrollment, such as guidance counselors, language teachers, and language department administrators” (Baggett, 2016, p. 175).

With these gaps in mind, the current study takes a large-scale longitudinal approach drawing on data from the Miami School Readiness Project (MSRP; Winsler et al., 2008, 2012, 2014; Serafini et al., 2020; Nguyen and Winsler, 2021). Previously, we examined foreign language learning and third language learning (L3) in the larger MSRP sample that included monolingual English students and students who spoke many other languages at home (*N* = 32,779; Nguyen and Winsler, 2021). There, we found that 59.4% of all students enrolled in some type of non-English language course, and with 47.7% enrolled in Spanish, and 19.9% enrolled in other non-English language courses. Here, we follow a subsample of low-income (*N* = 17,341) Spanish-speaking heritage students—those for whom parents reported Spanish to be their primary home language at school entry (kindergarten or 1st grade)—from age four through high school. Here, we investigate: (a) what percentage of heritage speakers of Spanish enroll in Spanish and other non-English language courses in middle and high school, (b) when and how long they took these courses, and (c) what demographic and early academic factors predict their enrollment and performance in Spanish courses. By providing a longitudinal look at Spanish heritage students’ secondary language study within a particular sociopolitical context, this work takes a significant step toward understanding the secondary school enrollment patterns of heritage students in Spanish (and other languages) and which factors predict their academic success in those courses.

The current study addresses the following questions. First, we ask the preliminary, descriptive (but still important) question of (1) At what rate do heritage speakers of Spanish enroll in Spanish and other non-English language courses in secondary school, and when, and for how long, do they take such language courses? Then we ask our primary research questions: (2) What factors predict Spanish heritage speakers’ enrollment in Spanish courses? and (3) what factors predict Spanish heritage speakers’ performance in Spanish (and other non-English language) courses?

## Materials and methods

### Context and participants

The sample in the current paper is drawn from the Miami School Readiness Project (MSRP; Winsler et al., 2008, 2014; Nguyen and Winsler, 2021). The MSRP is a cohort-sequential, longitudinal study that followed five cohorts of 4-year-old children from school entry until the end of high school. The first of the five cohorts entered kindergarten in 2002, and the last in 2007. The children in the study were either enrolled in public school pre-K programs or qualified to receive childcare subsidies for low-income families at age 4 (Winsler et al., 2008). In the year before each cohort of children entered kindergarten, school readiness assessments were administrated to evaluate their cognitive, language, socio-emotional, and motor skills. School information (including grades, courses taken, and standardized test scores) was collected every year (Winsler et al., 2008, 2012, 2014).

For the purpose of the current study, the sample included only students for whom (a) Spanish was listed as their home language at school entry (as reported by parents), (b) home language data were present in kindergarten or first grade, and (c) school transcript data were available in 6th grade or later. Our total sample was *N* = 17,341 heritage speakers of Spanish (see Table 1). We use data obtained through academic year 2016–2017, during which some students in our sample were still completing high school. Cohort A and B had completed 12th grade (*n* = 2,838 [16.4%]; and *n* = 3,413, [19.7%], respectively), while the other three cohorts had only completed 11th grade (cohort C; *n* = 3,924; 22.6%), 10th grade (cohort D; *n* = 3,871; 22.3%), or 9th grade (cohort E; *n* = 3,295; 19%). Students who were retained in grade (repeated a year) were also included in the sample.

**Table 1:** Demographic.

**Total sample**	*N* = 17,341
Has 6th grade data	*n =* 16,738
Has 7th grade data	*n =* 16,550
Has 8th grade data	*n =* 15,922
Has 9th grade data	*n =* 13,408
Has 10th grade data	*n =* 10,250
Has 11th grade data	*n =* 6,032
Has 12th grade data	*n =* 2,623
**Gender**	
Male	9,186 (53%)
Female	8,155 (47%)
**Ethnicity**	
Hispanic	16,538 (95.4%)
Black	341 (2%)
White/Asian/other	462 (2.7%)
**Poverty status (6th grade)**	
Received free/reduced-price lunch	13,886 (82.8%)
Did not receive free/reduced-price lunch	2,890 (17.2%)
**Disability status (6th grade)**	
Has a disability	3,004 (18.3%)
Non-disabled	13,438 (81.7%)
**School readiness skills (nat. percentiles)**	*M (SD)*
*LAP-D (1–99 scale)*	
Cognitive skills	50.12 (29.79)
*DECA (1–99 scale)*s	
Socio-emotional skills	59.69 (27.55)
Behavioral concerns	44.58 (29.13)
**5th grade elementary academic achievement**	
FCAT math (1–5 scale)	2.16 (1.32)
FCAT reading (1–5 scale)	2.17 (1.29)
GPA (0–4 scale)	3.25 (0.51)

The current study took place in Miami Dade County, Florida, United States. This is a linguistically and ethnically diverse area, with 70% Hispanic/Latino, 17% Black/African American, 14% White, and 75% reported speaking a language other than English at home (U.S. Census Bureau, 2020). Notably, in schools, the dominant instructional language is English, despite the prevalence of Spanish in the environment (66.3% of the population report speaking Spanish at home; U.S. Census Bureau, 2020). In the current context, there were various bilingual education programs offered in elementary school, varying from transitional bilingual education models (i.e., Mainstream-Inclusion Core/Basic Subject Areas, Mainstream-Inclusion English/Language Arts, Sheltered Core/Basic Subject Areas, Sheltered English/Language Arts) to DLI programs (i.e., One-Way Immersion, Dual Language or Two-Way Immersion; Serafini et al., 2022); however we do not have child-level data on who experienced which type of bilingual education in elementary school. In terms of the type of Spanish elective courses offered during secondary school, we know that the school system offered both “Spanish 1, 2, 3” courses as well as “Spanish for Spanish Speakers” courses. Unfortunately, we do not have child-level data on which type of Spanish course the heritage speakers in our study took. However, based on analyses for the entire MSRP sample – which includes English monolinguals and speakers of other languages (Nguyen and Winsler, 2021), we know that 55% of all students who took any type of Spanish courses were heritage speakers of Spanish. Further, group-level preliminary analyses show about 23% of all high school students studying were enrolled in a “Spanish for Spanish Speakers” course; at the middle school level, 40% of students studying Spanish took Spanish courses designed for Spanish speakers. Thus, we estimate that about half of the current sample of heritage Spanish speakers took heritage language courses designed for Spanish speakers.

Students classified as dual language learners by the public-school system receive English for Speakers of Other Languages (ESOL) services and must complete a yearly English proficiency test. Once students reach the highest ESOL level determined by the school system (5), they are considered English proficient and exit the program. Thus, our sample consists of students with varying degree of proficiency in English across different elementary school years but who are all proficient in English by secondary school.

Finally, students in our sample have sufficient access to non-English language courses in secondary school, with 92.5% of middle schools and 100% of high schools in the study offering Spanish (and other) language courses (Nguyen and Winsler, 2021). According to the school system, foreign language is not required for students to graduate high school with a ‘standard’ diploma so it is technically correct for us to use the term ‘elective’ language courses when speaking about these language courses. However, 2 years of high school foreign language classes (or demonstration of foreign language proficiency on a test at a level equal to 2 years of high school foreign language) is required for several of the more advanced college prep diploma types, and is required for application to 4-year state universities (Miami-Dade County Public Schools, n.d.).

### Measures

#### Language learning outcomes

The MSRP tracked five cohorts of students from 2002 to 2016. The first cohort of students reached middle school (6th grade) in 2009. By 2013, all five cohorts had reached 6th grade and were presented with the opportunity to take “foreign language” courses. Students can, of course, take non-English language courses multiple times across secondary school. Thus, in the present study, students’ enrollment and performance in these classes were determined at each grade level and then combined to create overall variables capturing if students had *ever* taken any Spanish and other non-English language courses, as well as their *average* performance in these courses across all instances/years of taking language courses (for details, see Nguyen and Winsler, 2021).

##### Enrollment

Students were coded for *Spanish enrollment* if they had ever taken a Spanish course any time between 6th and 12th grade (1 = yes, 0 = no). Students were coded for *other non-English language enrollment* if they had ever taken a language course in another non-English language any time between 6th and 12th grade (1 = yes, 0 = no). However, as explained above, it is important to note that we do not have information about what the Spanish courses were called. Other foreign language courses that appeared on the transcripts included French, German, Chinese, Russian, Latin, Italian, Greek, Japanese, Portuguese, and Hebrew.

##### Performance

Students received a grade for each language course they took (original performance obtained in ordinal letter grades—A, B, etc. and converted to a 0–4 scale). Grades across all language courses were averaged to create an overall, roughly continuous, performance variable. Students’ overall averaged performance across all Spanish courses and across all other non-English language courses was calculated between 6th and 12th grade.

#### Student-level predictors

##### Demographics

Demographic information was obtained using school records. The variables of interest include: gender (1 = male, 0 = female), students’ free/reduced-price lunch status (a proxy for poverty 1 = yes, 0 = no), and disability status (1 = student has at least one of the following exceptionalities according to the district: autism, visual impairment, deafness, brain injury, learning disability, intellectual disability, speech/language disorder, emotional disturbance, or other health impairment; 0 = no; See Table 1). Notably, in addition to Hispanic students (95.4%) whose parents reported the home language is Spanish, the current sample also included a small number of Black, White, Asian, and other race/ethnicities (4.7%) who reported a home language of Spanish. Ethnicity is reported in Table 1 for demographic purposes, but due to (a) very small numbers of White, Asian, and Black individuals in this heritage Spanish sample, and (b) the fact that race was unrelated to the outcomes of interest, and results for the other variables did not change when race was included/excluded in models, race/ethnicity was not included in the regression models.

##### School readiness

Children were assessed for school readiness at age 4. Specifically, *cognitive skills* were measured by the cognitive subscale The Learning Accomplishment Profile-Diagnostic (LAP-D; Nehring et al., 1992) at the beginning (September/October) and end (April/May) of pre-kindergarten year. The LAP-D is a national norm-referenced developmental assessment, reliable and valid for diverse populations, with four domains: cognitive, language, fine motor, and gross motor (Winsler et al., 2008). Assessments were given in either English or Spanish based on child’s strongest language as determined by the assessor and their teachers, and children were assessed individually (Winsler et al., 2008). Percentile scores from the cognitive subscale were used to measure children’s cognitive skills at age 4.

In addition, parents and preschool teachers filled out the Devereux Early Childhood Assessment (DECA; LeBuffe and Naglieri, 1999) at the beginning and end of the pre-kindergarten year to measure children’s *socio-emotional skills* and *behavior problems*. Higher scores for the subscales correspond to better socio-emotional skills and more behavior problems, respectively. The DECA is a nationally standardized assessment available in English and Spanish and is frequently used to measure socio-emotional skills in early childhood (Stewart-Brown and Edmunds, 2003). The DECA has 37 items in four subscales: initiative, attachment, self-control, and behavior concerns. DECA scores were determined as two main constructs: total socioemotional protective factors (TPF; 27 combining the initiative, attachment, and self-control scales) and behavioral concerns (10 items). Notably, the scale retains its integrity in linguistically and ethnically diverse and low-income children, which is important for the current sample of interest. In the MSRP, internal consistency alpha ranges from 0.71 to 0.94 (Crane et al., 2011) and does not vary by language of form (English, Spanish; see Nguyen and Winsler, 2021).

##### Prior/elementary school academic achievement

Prior academic achievement consists of 5th grade standardized test scores and teacher-assigned letter grades (grade point average [GPA]) in 5th grade. Students completed the high-stakes, state-wide, Florida Comprehensive Assessment Test (FCAT) beginning in third grade to assess achievement in reading and math (Florida Department of Education, 2019a). Both a standard score and a proficiency category were given to students, with proficiency ranging from 1 (little success with the challenging content) to 5 (success with the most challenging content). In the 2010–2011 school year, the state changed the test from the FCAT to the very different FCAT-2 (Florida Department of Education, 2019a). Thus, students in our sample would have taken only one of these tests in 5th grade but since this study includes 5 cohorts, some took different versions of the test. Due to this discrepancy, FCAT proficiency ordinal scores (1–5 scale) were included in our analyses instead of the standard scores (which were on different scales; Florida Department of Education, 2019a). The FCAT (English) reading score in fifth grade was used as a covariate measure of prior academic language performance. In addition, we conducted additional analyses replacing the reading score with the math score. Theoretically, the FCAT math score would demonstrate student general ability, while the reading score would be more influenced by student English language skills (for further details, see Nguyen and Winsler, 2021). Finally, 5th grade GPA consist of the overall average teacher-assigned letter grades across all subject areas (converted into a 5-point scale: 4.0 = A, 3.0 = B, 2.0 = C, 1.0 = D, 0.0 = F). Student GPA in 5th grade was used as a covariate measure of overall prior academic performance.

### Analyses

Descriptive statistics were used to answer the first question which focused on the percentage of heritage speakers who enrolled in Spanish and other non-English language courses, as well as the timing and length of that enrollment. For the last two questions, which investigated predictors of heritage speakers’ enrollment and performance in language classes, data were analyzed using hierarchical multiple regression (logistic regression for enrollment, linear regression for performance). The first block included demographic variables (gender, poverty, and disability status); the second block included early school readiness skills at age 4 (cognitive skills, social skills, and behavior concerns); and the final block included prior academic achievement (fifth grade GPA, and test scores).

#### Missing data

Due to the longitudinal and school-based, real-world nature of the study, there were missing data on some predictors as well as attrition in the sample over time. Since our inclusion criteria required that students have at least some 6th grade or later data, and all students had a chance to reach 9th grade based on their age/cohort, we defined longitudinal attrition as middle school students who did not have any high school data (grade 9 or above) meaning that the student would have left the public school system before 9th grade and did not return. Across the full sample, 14.2% did not have any high school data (left the public school system). Given the large sample size, we only note correlations between missingness and relevant variables that are greater than *r* = 0.10. Missing high school information moderately correlated with several predictors and outcomes, including disability (*r* = −0.11), FCAT reading and math (*r* = 0.35 and *r* = 0.34, respectively), Spanish enrollment (*r* = 0.20), Spanish and other non-English language course grade (*r* = 0.19 and *r* = 0.12, respectively). In sum, students missing high school data were less likely to have a disability and had higher initial achievement (in general and for middle school Spanish/language classes) than students who remained in the sample.

Missing data for predictors were less than 17% of cases, with the exception of cognitive skills (39.7% missing) and 5th grade GPA (23.7% missing). Some correlations between the missingness on predictors and the outcome were moderate, including disability (*r* = 0.30), cognitive skills (*r* = −0.24), social skills (*r* = −0.14), FCAT reading and math (*r* = −0.30 and *r* = −0.29, respectively), Spanish enrollment (*r* = −0.20), and Spanish and other non-English languages performance (*r* = −0.20 and *r* = −0.13, respectively). Thus, students missing a predictor variable were more likely to have a disability, tended to score lower on school readiness skills, and were less likely to take (and performed poorer in) Spanish and other language courses compared to students with no missing predictors.

We first ran the set of analyses described above using listwise deletion in IBM SPSS Statistics, then conducted additional analyses in R (https://www.R-project.org/) using the lavaan package (Rosseel, 2012) to use full information maximum likelihood (FIML) to adjust for missing data on the predictors.

## Results

### RQ1: Enrollment in Spanish and other non-English language courses

The first research question was answered using descriptive statistics. Frequencies were used to analyze the number of students taking Spanish and other non-English language courses in each grade level (Table 2). Of the 17,341 heritage Spanish speakers in our sample, 11,414 (65.8%) enrolled in some type of language course at least once from grade 6 to grade 12. More specifically, 52.2% enrolled in Spanish courses and 25.3% enrolled in other non-English language courses; it should be noted that these categories are not mutually exclusive, as some students took both types of language courses. In general, the percentage of enrollment in heritage Spanish courses is higher, sometimes over twice the percentage of students enrolling in other non-English language courses. In terms of enrollment patterns, within the 11,414 students mentioned above who took some type of language course in secondary school, 61.5% (*n* = 7,019) of heritage speakers only enrolled in Spanish courses, 20.8% (*n* = 2,368) only enrolled in other non-English language courses, and 17.7% (*n* = 2,012) enrolled in both types of courses in secondary school.

**Table 2:** Enrollment in non-English language courses of heritage Spanish students courses in secondary school by year and type of course.

	Ever in middle school		Ever in high school		Ever in secondary school		Grade 6		Grade 7		Grade 8		Grade 9		Grade 10		Grade 11		Grade 12	
Total	17,178		13,567		17,329		16,738		16,550		15,922		13,408		10,250		6,032		2,623	
Type of course	*N*	%	*N*	%	*N*	%	*N*	%	*N*	%	*N*	%	*N*	%	*N*	%	*N*	%	*N*	%
Any non-English	5,667	33%	9,485	69.9%	11,414	65.8%	3,413	20.4%	3,472	21%	3,486	21.9%	4,958	37%	5,973	58.3%	3,257	54%	1,239	47.2%
Spanish	4,242	24.7%	7,137	52.6%	9,043	52.2%	2,351	14%	2,397	14.5%	2,573	16.2%	3,241	24.2%	4,178	40.8%	2,370	39.3%	977	37.2%
Other non-English/Spanish	2,034	11.8%	3,060	22.6%	4,383	25.3%	1,354	8.1%	1,331	8%	1,088	6.8%	1,779	13.3%	1,881	18.4%	917	15.2%	274	10.4%

Course categories are not mutually exclusive; a student can enroll in both Spanish and other non-English/Spanish courses. Percentage is calculated using the total sample for that grade/time period.

Table 3 depicts the grade at which heritage Spanish students first took a non-English language course. Among heritage Spanish speakers enrolled in non-English languages courses in secondary school, most students first took Spanish in sixth (27%), ninth (20.1%), or tenth (21.8%) grade. Similarly, most students who took other non-English languages courses also began in these grades (32, 28.3, and 18.4% respectively). Sixth grade is the first grade of middle school and 9th grade the first year of high school in this district, suggesting that students tend to take heritage language courses most when they enter the next level of schooling, and it is less common to start taking languages later.

**Table 3:** The grade at which heritage Spanish students first took a non-English language course.

Grade	Spanish course		Other non-English/Spanish courses	
	N	%	N	%
6	2,351	27.0%	1,354	32.0%
7	1,028	11.8%	436	10.3%
8	800	9.2%	226	5.3%
9	1750	20.1%	1,197	28.3%
10	1893	21.8%	779	18.4%
11	694	8.0%	214	5.1%
12	184	2.1%	29	0.7%
Total	8,700		4,235	

In terms of Spanish enrollment patterns, of the heritage Spanish speakers who took some type of Spanish course, 14.6% (*n* = 1,208) enrolled only in middle school, 57.2% (*n* = 4,723) enrolled only in high school, and 28.2% (*n* = 2,335) enrolled at least once in both middle school and high school. Similarly, of the students who took some type of other non-English language course, 23.7% (*n* = 944) enrolled only in middle school, 58.4% (*n* = 2,321) enrolled only in high school, and 17.9% (*n* = 711) enrolled in both middle school and high school.

Table 4 shows the total number of grades in which students took non-English language courses. Of the 9,043 heritage students who took Spanish courses, most students only enrolled in one (39.2%) or two (34.9%) Spanish courses/years. Of the 4,383 heritage students who took some type of other non-English language course, the largest group of students also only enrolled in one (43.8%) or two (32.5%) other non-English language courses/years. Interestingly, a small number of students enrolled in these courses for all 7 years of secondary school (23 and 20 in Spanish and other non-English language courses, respectively).

**Table 4:** Total number of grades heritage Spanish students took non-English language courses.

Number of grades taken	Spanish courses		Other non-English/Spanish courses	
	N	%	N	%
1	3,545	39.2%	1,918	43.8%
2	3,158	34.9%	1,424	32.5%
3	1,491	16.5%	598	13.6%
4	594	6.6%	255	5.8%
5	176	1.9%	104	2.4%
6	56	0.6%	64	1.5%
7	23	0.3%	20	0.5%
Total	9,043		4,383	

### RQ2: What factors predict heritage Spanish speakers’ enrollment in Spanish courses?

For this question, a hierarchical logistic regression was conducted with the entire sample (*N* = 17,341). Table 5 shows the results where odds ratios (OR) are provided. An OR greater than 1 indicates an increase in the odds of taking Spanish courses, and an OR less than 1 indicates a decrease in the odds of taking Spanish courses. For categorical variables, the OR is a function of being on one level of the variable (i.e., male) compared to the other (female). For continuous variables, OR indicates the increase/decrease in odds of FL enrollment with a 1-point increase in the predictor.

**Table 5:** Logistic regression predicting Spanish enrollment in secondary school (*n* = 7,382).

	Model 1		Model 2		Model 3	
	*OR*	*SE(B)*	*OR*	*SE(B)*	*OR*	*SE(B)*
**Demographics**						
Male	0.960	0.049	0.995	0.050	1.063^c^	0.051
Lunch (poverty)	0.712***	0.068	0.747***	0.069	0.848*	0.070
Special education	0.504***	0.104	0.552***	0.106	0.671***	0.108
**School readiness at age 4**						
LAP-D cognitive skills			1.002	0.001	1.000^c^	0.001
DECA social skills			1.001	0.001	1.000	0.001
DECA behavior concerns			0.997**	0.001	0.998*	0.001
**Elementary academic performance**						
GPA in 5th grade					1.535***	0.055
^a^Reading 5th grade					1.144***	0.020
^a^Math in 5th grade					1.155***	0.020

*p < 0.05, **p < 0.01, ***p ≤ 0.001. ^a^Math and reading scores were run in different models to avoid multicollinearity. ^c^Results are significant when analyzed with FIML.

Overall, in model 1 with only demographic predictors, poverty status and disability status, but not gender, uniquely predicted Spanish courses enrollment. Specifically, poverty appeared to hinder heritage speakers’ enrollment in Spanish courses, as those who receive free or reduced-price lunch had lower odds of taking a Spanish course than those who did not (OR = 0.712, *p* < 0.001). Similarly, heritage speakers with disabilities had significantly lower odds of Spanish course enrollment compared to those without disabilities (OR = 0.504, *p* < 0.001).

In model 2, when student school readiness skills were added, only early behavior concerns predicted later Spanish course enrollment for heritage speakers. A one-point increase in behavior concerns at age 4 was associated with 0.003 decrease in enrollment odds. In other words, a student in the 50th percentile compared to a student in the 25th percentile (a 25-point difference) in behavior problems at school entry would have a 7.5% increased chance (25 × 0.003) of enrolling in Spanish courses in secondary school. Poverty and disability status remained significant negative predictors even after controlling for school-entry skills.

In the last block, student 5th grade achievement was entered. GPA and test scores in 5th grade significantly predicted the odds of taking at least one Spanish course in middle or high school. A 1-point increase in GPA (e.g., moving from a C to a B) increased the odds of enrolling in Spanish courses by 53.5%. Similarly, a 1-point increase in test scores (on a 1–5 point scale) increased the odds of enrollment by about 15% for both math and reading. Interestingly, lunch status, disability status, and early behavior concerns remained significant. Additional analyses were conducted with a maximum likelihood estimator to account for missing data. Findings remained similar, with only minor differences. Poverty status was no longer a significant predictor. In the model with FCAT reading, cognitive skill became a significant predictor (*OR* = 1.000, *p* = 0.016); in the model with FCAT math, gender was a significant predictor (*OR* = 0.978, *p* = 0.003). Overall, heritage students who had a disability or had more behavior problems were less likely to enroll in Spanish than their counterparts. In addition, those with higher prior GPAs and standardized test scores were more likely to enroll in Spanish than those with lower grades and scores.^1^

### RQ3: What factors predict heritage Spanish speakers’ performance in language courses?

#### Spanish language course performance

For this research question, two hierarchical multiple regression analyses were conducted for each language type (Spanish vs. other non-English language courses). Table 6 shows results for the analyses concerning Spanish course performance. The model significantly predicted average performance (GPA) across all Spanish courses taken by students, *F*(3,4,656) = 109.396, *p* < 0.001, *R*^2^_adjusted_ = 0.065. On average, male students, students in poverty, and students with a disability had lower grades than their counterparts (*B*_male_ = −0.349, *B*_lunch_ = −0.245, and *B*_disability_ = −0.375, all *p* < 0.001, respectively).

**Table 6:** Multiple regression predicting Spanish course performance in secondary school (*n* = 4,660).

	Model 1			Model 2			Model 3		
	*B*	*SE(B)*	*β*	*B*	*SE(B)*	*β*	*B*	*SE(B)*	*β*
**Demographics**									
Male	−0.349***	0.024	−0.207	−0.322***	0.024	−0.191	−0.239***	0.023	−0.142
Lunch (poverty)	−0.245***	0.031	−0.112	−0.197***	0.031	−0.090	−0.084**	0.029	−0.038
Special education	−0.375***	0.061	−0.088	−0.286***	0.061	−0.067	−0.077^c^	0.058	−0.018
**School readiness at age 4**									
LAP-D cognitive skills				0.003***	0.000	0.093	0.000	0.000	0.016
DECA social skills				0.001*	0.001	0.033	0.000	0.001	0.006
DECA behavior concerns				−0.002***	0.000	−0.066	−0.001^c^	0.000	−0.028
**Elementary academic performance**									
GPA in 5th grade							0.660***	0.027	0.367
^a^Reading 5th grade							0.005	0.009	0.008
^a^Math in 5th grade							0.009	0.009	0.014
*R* ^2^	0.065			0.083			0.196		
*R*^2^ change				0.018***			0.113***		

*p < 0.05, **p < 0.01, ***p ≤ 0.001. ^a^Math and reading scores were run in different models to avoid multicollinearity. ^c^Results are significant when analyzed with FIML.

In step 2, early school readiness skills were added to the model, and the model significantly predicted student performance in Spanish language courses (*F*(6,4,653) = 71.56, *p* < 0.001, *R*^2^_adjusted_ = 0.083), and significantly improved the prediction compared to model 1 (*R*^2^_change_ = 0.018, *p* < 0.001). In general, students with higher cognitive and socials skills at age 4 outperformed students with lower scores in these skills 7+ years later (*B* = 0.003, *p* < 0.001; and *B* = 0.001, *p* = 0.043, respectively). In addition, students with lower behavior problems in preschool had higher average scores in Spanish courses compared to those with higher early behavior concerns (*B* = −0.002, *p* < 0.001). Demographic factors remained significant.

The last model included the influence of 5th grade achievement on secondary school Spanish course performance. Model 3 significantly predicted students’ performance in Spanish courses (*F*(8, 4,651) = 413.297, *p* < 0.001, *R*^2^_adjusted_ = 0.196), above and beyond compared to the previous model (*R*^2^_change_ = 0.113, *p* < 0.001). Notably, GPA (*B* = 0.66, *p* < 0.001), but not math or reading scores predicted Spanish language performance for heritage Spanish students, such that higher 5th grade GPA was associated with better grades later in Spanish classes in secondary school. Interestingly, only gender and poverty status remained significant predictors in this model with 5th grade performance added. The same analyses were conducted with FIML to account for missing data. Findings remained similar, although disability status (*b* = −0.103, *p* = 0.001) and behavior concerns (*b* = −0.001, *p* = 0.032) became significant predictors as well. Overall, male students, students in poverty, students with a disability, and those with more behavior problems than their peers had lower grades in Spanish courses. In addition, students with a higher GPA in 5th grade displayed higher Spanish course performance.

#### Other non-English language course performance

Similar patterns were found for other non-English/Spanish language course performance across secondary school. Overall, model 1 significantly predicted performance and all the same predictors were associated with student grades in other non-English courses. In model 2, only cognitive skills and behavior concerns predicted other non-English/Spanish language course performance—children with higher cognitive skills and lower behavior concerns at age 4 achieved higher grades in other non-English language courses over 7 years later. However, in model 3, school readiness skills were no longer significant predictors. Similar to findings about Spanish courses, only gender and poverty status remained significant demographic predictors in this model. Notably, unlike the findings about Spanish performance where only GPA was a significant predictor, GPA and test scores (*B*_reading_ = 0.048, *B*_math_ = 0.44, all *p* < 0.001) significantly predicted performance in other non-English language courses, with higher grades and test scores associated with higher grades in these courses. The final model accounted for 25.5% of variance in other non-English language course performance of heritage Spanish students (Table 7). Additional analyses were conducted using FIML to account for missing data. Findings remained the same. Overall, male students and students in poverty had lower grades in other non-English language courses compared to their counterparts, and students with higher 5th-grade achievement performed better in these courses.

**Table 7:** Multiple regression predicting other non-English language course performance in secondary school (*n* = 2,325).

	Model 1			Model 2			Model 3		
	*B*	*SE(B)*	*β*	*B*	*SE(B)*	*β*	*B*	*SE(B)*	*β*
**Demographics**									
Male	−0.470***	0.043	−0.219	−0.429***	0.043	−0.200	−0.294***	0.040	−0.137
Lunch/poverty	−0.410***	0.057	−0.145	−0.330***	0.057	−0.116	−0.136*	0.053	−0.045
Special education	−0.369**	0.127	−0.058	−0.251***	0.127	−0.040	−0.057	0.115	−0.009
**School readiness at age 4**									
LAP-D cognitive skills				0.004***	0.001	0.118	0.001	0.001	0.023
DECA social skills				0.002	0.001	0.046	0.000	0.001	0.010
DECA behavior concerns				−0.002*	0.001	−0.048	0.000	0.001	−0.009
**Elementary academic performance**									
GPA in 5th grade							0.988***	0.048	0.419
^a^Reading 5th grade							0.048***	0.015	0.058
^a^Math in 5th grade							0.044***	0.017	0.056
*R* ^2^	0.073			0.094			0.255		
*R*^2^ change				0.021***			0.161***		

*p < 0.05, **p < 0.01, ***p ≤ 0.001. ^a^Math and reading scores were run in different models to avoid multicollinearity.

## Discussion

To the knowledge of the authors, the current paper is the first of its kind to longitudinally explore the language study of heritage Spanish speakers in Spanish or non-English language courses in secondary school at a large scale in the United States. We found that the predominantly low-income, heritage Spanish speakers in this community enrolled in language courses at a high rate in every grade (20.4–58.3%) and in total (66%) in secondary school, and heritage speakers enrolled in Spanish at almost twice the rate as other non-English language courses. Nationally for all K-12 students, the estimate is that only about 20% of students take non-English language courses in school (American Councils for International Education, 2017) in a given school year. This level of interest in learning non-English languages is notable and likely reflects not only serious commitment on the part of the students to maintain Spanish and master an L3, but also demonstrates the dedication of the school system, within this context, to provide students with opportunities to access and pursue language learning throughout the secondary years. Given that 2 years of second language courses is required in high school for students to receive the more advanced, college bound diploma types, these high rates of language course enrollment may also reflect high educational aspirations for Spanish heritage students.

The preference to study Spanish observed here is also consistent with national trends showing that enrollment in Spanish is more than three times the total enrollment in other major languages including French, Arabic, Chinese, German, Japanese, Latin, and Russian (American Councils for International Education, 2017). Enrollment patterns varied across grade levels and types of language, with some students taking multiple languages or continuously enrolling in the same type of languages (Spanish or other non-English languages) over several years. Our findings also provide large-scale longitudinal evidence as to which student background variables significantly predict enrollment and performance in these language courses. Overall, findings ssp contribute to better understanding the broader picture of Spanish-speaking heritage students’ language enrollment patterns in secondary school. Moreover, the current research has key implications for understanding and supporting heritage students’ continued language learning and long-term language maintenance, and the crucial importance of offering equal opportunities for students to access heritage courses specifically designed to meet their needs.

### Enrollment of heritage speakers in non-English language courses

To date, there has been little research on the non-English language course-taking of Spanish heritage language speakers at the secondary school level in the United States. Indeed, prior research primarily focused on students in higher education (Potowski, 2002; Brown and Thompson, 2018; Looney and Lusin, 2019; Nagano et al., 2019), even though evidence suggests that heritage learners’ proficiency in high school positively predicts college academic attainment (Jang and Brutt-Griffler, 2019), and starting language courses earlier (i.e., middle school as opposed to high school) leads to higher motivation for language learning in students (Kissau et al., 2015). There are no current national statistics regarding the rate of enrollment for heritage students in Spanish or other non-English language courses, even though this is one of the fastest growing groups of students in the U.S. (National Center for Education Statistics, 2018).

In the current sample, 65.8% of Spanish heritage students enrolled in some type of language courses in secondary school; within this group, around 62% enrolled in Spanish, 20% enrolled in other non-English language courses, and 18% enrolled in both types of language courses. Notably, the rate of general non-English language course enrollment is slightly higher in heritage students compared to the larger sample of students in the MSRP (*n* = 33,247, 59.4%), especially in the rate of Spanish enrollment (47.7% in full MSRP sample, 62% in the heritage student sample; Nguyen and Winsler, 2021). This is consistent with prior research suggesting that students who speak multiple languages may be more inclined to enroll in additional language courses at the secondary school level (Nguyen and Winsler, 2021). Although we do not know which type of Spanish courses individual students took, we know that about 30% of the courses were “Spanish for Spanish Speakers” classes designed specifically to meet the needs of heritage speakers. These are promising numbers as heritage students are known to experience a range of benefits from home language study in heritage language courses, including ethnolinguistic pride, heritage language maintenance, and increased student motivation and persistence/retention (Carreira, 2000; Leeman et al., 2011; Amezcua, 2019; Prada et al., 2021; Serafini, 2021; Holguín Mendoza, 2022).

In addition to general enrollment rates, we explored the timing of enrollment in Spanish and other non-English courses for Spanish heritage students. Students usually began enrollment at the beginning of middle school (6th grade), or the first 2 years of high school (9th and 10th grades). It is possible that the bump seen in language course enrollment at the beginning of middle and high school is due to guidance counselors emphasizing the importance of language courses during these school transitions. Notably, across all types of language courses, most students enrolled in language courses in high school rather than middle school, with a sizeable minority enrolled continuously in middle school and high school. Given that college entrance sometimes requires demonstration of other language proficiency, perhaps students are encouraged to begin taking language courses at the high school level given its relevance, in line with national trends and prior research showing greater language enrollment in high school than in middle school (Pufahl and Rhodes, 2011; American Councils for International Education, 2017; National Center for Education Statistics, 2018; Nguyen and Winsler, 2021).

Heritage students who enrolled either in Spanish or other non-English language courses in middle school might have a higher level of interest and motivation in maintaining and improving their home language or learning novel languages given their experience being bilingual (Nguyen and Winsler, 2021). This “cyclical bilingualism,” in which adolescents seek to reacquire or further develop the heritage language(s) they spoke in childhood, is well documented (Silva-Corvalán, 1994; Villa and Rivera-Mills, 2009). In addition, those who enrolled in language courses in both middle school and high school may be uniquely different than their peers, and more likely to experience both short and long term cultural and social advantages associated with the continued pursuit of language learning (Wight, 2015). When students did take language courses, they usually enrolled for one to 2 years rather than longer, and very few students enrolled for as long as 7 years. This is reasonable as students also must take many other subjects across the middle school and high school years; as language courses are usually electives, they may not be high on the priority of courses to be taken continuously, unlike math and literature. Indeed, most states do not require foreign language study for graduation (AICE, 2017; Met and Brandt, 2017), and language courses were not mandatory in the state of Florida at the time of the present study (Florida Department of Education, 2019b).

#### Predicting heritage speakers’ enrollment in Spanish courses

In addition to enrollment patterns in Spanish and other language courses, we explored student-specific factors that may predict heritage speakers’ enrollment in Spanish courses. Findings reveal that students in poverty, students who had a disability, and students who displayed more behavior problems early on had a lower likelihood of enrolling in Spanish courses compared to their peers, while those with better elementary school achievement were more likely to take these courses. Indeed, students in poverty may not consider elective language courses to be a high priority as they are faced with additional responsibilities in the household or experience additional stressors that prevent them from devoting time to schoolwork (Jensen, 2009). Similarly, students with a disability may actively choose not to take language courses even when it is their home language due to the belief that they lack the academic skills to succeed and feel less positive about language learning despite wanting to learn (Sparks et al., 1993), and they may also be discouraged to enroll in them due to false assumptions of teachers and counselors about students’ abilities (Sparks, 2016). Sustained opportunities to build literacy in and maintain one’s home language can be framed not only as an individual ‘right’, but also as contributing to strengthening a collective ‘resource’ (Peyton et al., 2001). Further, language courses are beneficial to all students as they can provide pragmatic, cognitive, and cultural gains (Sparks, 2016); thus, educators have an ethical responsibility to encourage heritage Spanish students with a disability to take Spanish courses and to advocate for more systematic heritage course offerings at all levels of education.

Another factor associated with a lower likelihood of Spanish enrollment for heritage Spanish speakers was greater preschool behavioral problems. Prior research conducted in the same population showed that behavior problems were linked to slower English attainment in dual language learners (Winsler et al., 2014) and poorer academic performance later on in general (Ricciardi et al., 2021). Relatedly, students with higher 5th grade GPA and math and reading test scores were more likely to enroll in Spanish courses than their peers, which is consistent with prior findings in the larger population about language course enrollment more generally (Nguyen and Winsler, 2021). Students who perform well in school may be more likely to be encouraged to take different kinds of electives including languages as ‘enrichment’ or they have more freedom to choose additional electives instead of having to take remedial courses and extra study halls as is often required for students struggling academically.

In sum, Spanish heritage students’ background and achievement appear to influence whether they enroll in Spanish courses, given that access to these courses is not a major issue (92.5% of middle schools and 100% of high schools in our sample offered foreign language courses including Spanish; Nguyen and Winsler, 2021). Our findings are consistent with prior research about general language learning for all students in the current context (Nguyen, 2020) and provide additional understanding for researchers and educators of the factors predicting heritage students’ Spanish course enrollment at the secondary level.

One important perspective for interpreting factors associated with enrollment is that access to Spanish classes and language maintenance are equity issues. The type of students who tend not to enroll in Spanish language courses are students who may lack necessary resources available to them (those in poverty, those with disabilities) and might be the most likely to benefit from such courses. It is well-documented that minority language populations such as Spanish heritage speakers are underserved within the U.S. education system due, at least in part, to a lack of resources. As previously discussed, a key obstacle is the gentrification of bilingual education models, particularly those that are known to be most effective in closing the ‘gap’ among language majority and language minority children (Serafini et al., 2022). That is, schools that do offer DLI programs have been critiqued for catering to the needs of non-Hispanic White students and students from English-speaking and/or affluent homes (Flores and García, 2017; Flores et al., 2021). Thus, certain groups of students in our sample may be disadvantaged in multiple ways, being a Spanish heritage speaker while also being in poverty or having a disability. It is crucial that educators be aware of these intersectional structural inequities to better serve these students.

### Performance of heritage speakers in non-English language courses

Beyond enrollment, we were also interested in performance of heritage students in both Spanish and other non-English language courses. Findings across the different language types are similar; male students, students in poverty, students with a disability, and students with more preschool behavior problems had lower grades than their counterparts, while those with higher 5th grade GPA outperformed their peers in language courses. This is consistent with enrollment findings as well as previous findings about language course performance in the same population including all students (Nguyen and Winsler, 2021). Certain demographic effects went away when 5th-grade achievement was included as predictors (i.e., poverty, disability, school readiness), suggesting that performance in elementary school was the strongest predictor of performance in secondary school language courses.

An interesting difference between the findings of Spanish performance and other non-English language performance lies in the effect of 5th-grade test scores. Specifically, standardized test scores did not predict heritage student performance in Spanish courses but did predict performance in other non-English language courses. It is possible that heritage students who are learning Spanish, their home language, in a classroom environment are more engaged and invested than when in other courses, and performance in Spanish is less linked to traditionally assessed skills such as math and reading. Another possibility is that Spanish language courses (or *Spanish for Spanish Speakers* courses) do not tap into the type of knowledge assessed in standardized high-stakes testing. Performance in courses for which students have little prior knowledge (such as other non-English languages for Spanish speakers) appears more associated with general learning abilities such as reading and math.

### Limitations, implications, and future directions

The current study filled a gap in the literature concerning factors that impact heritage student enrollment and performance in Spanish or non-English language courses in middle school and high school. A limitation, however, is that the findings may not generalize to other settings and populations outside of Miami that have different ethnicity distributions and less ethnolinguistic vitality and sociolinguistic support in the community for Spanish language use and maintenance. We also lacked child-level information about whether students took “foreign language” Spanish courses vs. courses designed specifically for heritage Spanish learners. In spite of these limitations, the current paper offers novel descriptive information on Spanish and other non-English course enrollment patterns in secondary school for heritage Spanish speakers, and new understandings of factors related to heritage students’ enrollment and performance in Spanish and other non-English language courses. In addition, our longitudinal study design provided rich, robust evidence which allowed us to characterize trends of Spanish enrollment over time in heritage students, which have not been investigated previously. While our findings clearly do not inform us as to *why* heritage Spanish speakers chose to take or not take Spanish courses, prior research suggested that the discrepancy between students’ knowledge and course design, as well as educator beliefs may contribute to heritage student course selections (Potowski, 2002; Beaudrie et al., 2014; Parra, 2020; Leeman and Serafini, 2021). Future qualitative studies are needed to enhance our understanding of the lived experiences of heritage Spanish speakers, and their decision-making processes and motivations when it comes to selecting elective courses in secondary school.

## Conclusion

Heritage Spanish students are among the fastest growing group of K-12 students in the United States. Supporting and maintaining their home language is important concern not only at an individual level, but as a societal level as well. In general, there is a lack of Spanish language courses and programs designed to meet the need of these students. Specifically, English is typically the only language of instruction in the school system (Cummins, 2005; Wiley and García, 2016; Ennser-Kananen and King, 2018; Fuller and Leeman, 2020), and DLI courses are not usually available or may have moved away from prioritizing the dynamic bilingualism and cultural identities of Latinx and other minoritized communities (Flores and García, 2017; Flores et al., 2021). Further, Spanish courses at the secondary level often come in the form of Spanish as a *foreign* language rather than *heritage* language (Potowski, 2002).

The current paper contributes to the literature by describing Spanish course taking among heritage Spanish-speaking students and identifying factors related to heritage student enrollment and performance in Spanish or other non-English language courses in middle school or high school. Overall, heritage students enrolled at high rates in Spanish and other non-English language courses, with enrollment patterns varying across the grade levels, similar to the general population of K-12 students in the United States. Notably, student background and early achievement can predict both enrollment and performance of heritage students in these language courses. Thus, student motivation and goals may not be the only important component that leads to Spanish heritage speakers’ choices to pursue advanced language study as is often assumed. Our findings emphasize the need to apply a critical lens to the individual and social implications of U.S. language education, and provide useful insights for informing language education policy and underscoring the need for more systematic efforts to advocate for the needs, rights, and resources of language minority students.

## Data availability statement

The data analyzed in this study are subject to the following licenses/restrictions: Data are not available upon request because they belong to the public school system. The data use agreement in play does not allow other researchers to access the data. Questions about this should be directed to AW awinsler@gmu.edu.

## Ethics statement

The studies involving human participants were reviewed and approved by the original participating school system’s Institutional Review Board (IRB; Miami-Dade County Public Schools IRB 09141-01) and the participating university’s IRB (George Mason University #477930-9). Written informed consent to participate in this study was provided by the participant’s legal guardian/next of kin.

## Author contributions

MN and AW conceived of the presented idea. Data were previously collected in AW’s laboratory. MN performed all analyses. ES and JL contributed to the background theory and framework as well as the parameters for data analysis. All authors discussed the results and contributed to the final manuscript.

## Conflict of interest

The authors declare that the research was conducted in the absence of any commercial or financial relationships that could be construed as a potential conflict of interest.

## Publisher’s note

All claims expressed in this article are solely those of the authors and do not necessarily represent those of their affiliated organizations, or those of the publisher, the editors and the reviewers. Any product that may be evaluated in this article, or claim that may be made by its manufacturer, is not guaranteed or endorsed by the publisher.

## Footnotes

^1^The same analyses were conducted to predict other non-English language course enrollment. Similar to analyses for Spanish courses, disability status and elementary academic achievement were significant predictors. However, poverty did not predict non-English language course enrollment. In addition, in the first two steps, gender was a significant predictor, such that male students were less likely to enroll in other non-English language courses than female students, a result not seen for Spanish enrollment.
